# Smuggling as the “key to a combined market”: British American Tobacco in Lebanon

**DOI:** 10.1136/tc.2008.025254

**Published:** 2008-09-18

**Authors:** R Nakkash, K Lee

**Affiliations:** 1 Department of Health Management and Policy, Center for Research on Population and Health, American University of Beirut, Beirut, Lebanon; 2 Centre on Global Change and Health, London School of Hygiene and Tropical Medicine, London, UK

## Abstract

**Objectives:**

To understand the strategy of British American Tobacco (BAT) and other transnational tobacco companies (TTCs) to gain access to the Lebanese market, which has remained relatively closed under monopoly ownership and political instability.

**Methods:**

Analysis of internal industry documents, local language secondary sources and industry publications.

**Results:**

TTCs have relied on legal and illegal channels to supply the Lebanese market since at least the 1970s. Available documents suggest smuggling has been an important component of BAT’s market entry strategy, transported in substantial quantities via middlemen for sale in Lebanon and neighbouring countries. TTCs took advantage of weak and unstable governance, resulting in uncertainty over the Regie’s legal status, and continued to supply the contraband trade despite appeals by the government to cease undermining its revenues. Since the end of the civil war in the early 1990s, continued uncertainty about the tobacco monopoly amid political instability has encouraged TTCs to seek a legal presence in the country, while continuing to achieve substantial sales through contraband.

**Conclusion:**

Evidence of the complicity of TTCs in cigarette smuggling extends to Lebanon and the Middle East where this trade has especially benefited from weak governance and chronic political instability. The regional nature of TTC strategy supports strong international cooperation under the Framework Convention on Tobacco Control to tackle the problem.

The Middle East has been targeted by transnational tobacco companies (TTCs) since the 1970s as a key emerging market because of its young and growing population. Arab countries have grown, from 127 million in 1970 to 335 million people in 2007. Around 20% of the population is aged 15–24 years,^1^ a proportion predicted to increase as the population increases to 600 million by 2050.^2^ Between 1990 and 1997, tobacco consumption in the region grew by 24.3%,^3^ making it “one of the few growing markets in the world for tobacco”.^4^ However, scholarly analysis of the tobacco industry in the Middle East remains limited, including study of industry efforts to expand sales.

Based on industry documents, this paper examines how TTCs have sought to gain market access to Lebanon. Lebanon’s relatively liberal attitudes and geographical location (bordered by Syria and Israel, and close to Cyprus) have made it a strategically important market. Smoking prevalence in 2002 was 46% for adult males and 35% for females,^5^ relatively high compared with 38% and 7%, respectively, in the Middle East and North Africa (MENA) region in 1995.^6^ The rising rate of young female smokers in Lebanon is also notable.^7^ Tobacco, in turn, poses a significant health burden in Lebanon, accounting for 3500 deaths annually.^8^

TTCs sought a major presence in the Lebanese market as early as the 1930s when a French company purchased assets of the Ottoman Regie to form the *Compagnie Libano-Syrienne des Tabacs*.^9^ Following the end of French rule in 1935, control was assigned to the French company with Lebanese shareholders.^10^ ^11^ This arrangement continued until the 1950s when the government returned the *Regie Libanaise de Tabacs et Tombacs* (hereafter the Regie) to state ownership, placing it under the tutelage of the Ministry of Finance in 1959, which exerted strict control of imports, exports and production.^12^ This was not intended as a permanent arrangement, with discussion of a planned tender in 1961.^13^ Periodic reviews of the Regie’s monopoly status have followed, with resolution hindered by the country’s political instability and sectarian fragmentation.

The Regie’s legal status has remained uncertain until the present day.^14^^–^16 A key hurdle has been the entanglement of the tobacco industry with key factions who historically grant favours to farmers and Regie employees in exchange for their political support. Equally important has been government support for tobacco farmers in southern Lebanon to keep them on land threatened by Israeli incursion. The government has also been keen to protect revenues earned from the tobacco industry. The goal of the Regie, which employs around 3800 individuals and affects the livelihoods of 40 000–50 000 people in the agriculture, industrial and commercial sectors,^17^ has been a profitable company that supplies the domestic market and competes internationally. This complex mix of competing interests has been complicated by the civil war (1975–90) and ongoing political instability.

Within this context, TTCs have sought access to this substantial and growing market. Local cigarette brands dominated the market until the 1970s (two-thirds of supply in 1970), after which imports by Philip Morris, British American Tobacco (BAT) and Japan Tobacco grew to 85% of the market by 2000.^18^ Imports have since grown from 5554 million sticks in 2000 to 9574 million sticks in 2006.^19^ This analysis shows that smuggling has played an important part in ensuring the supply of foreign brands to Lebanon. TTCs have long denied involvement in the contraband trade,^20^ which globally accounted for around 10.7% of sales in 2005.^21^ However, previous analysis of industry documents suggests TTC complicity in Europe,^22^ ^23^ Asia,^24^^–^26 North America^27^ and Latin America.^28^ In 2003–4, the Eastern Mediterranean Regional Office (EMRO) of the World Health Organization published two reports of cigarette smuggling in the region, focused on Iran and Iraq, based on industry documents accessible at that time.^29^ ^30^ Enabled by improved public access, this paper undertakes a detailed analysis of TTC activity in Lebanon. Documents suggest that contraband was used by TTCs to build market share, as well as being a means of furthering regional expansion. A fuller understanding of this strategy supports the need to implement the WHO Framework Convention on Tobacco Control (FCTC) ratified by Lebanon in 2005, and negotiation of an FCTC protocol on cigarette smuggling.

## METHODS

This paper is based on industry documents obtained online from the British American Tobacco (BAT) Document Archive (BATDA) and Legacy Tobacco Documents Library (LTDL), and on-site from the BAT Guildford Depository. The obstacles to accessing, and limitations of working with, BAT documents have been described elsewhere.^31^^–^34 Based on an iterative search strategy, an on-site search at file level was undertaken in 2004 of the depository’s crude index using keywords of place and organisational names related to the Middle East. A total of 65 files were reviewed.

Online searches at document level were undertaken between 2004 and 2007 of LTDL and BATDA using keywords generated from initial searches and secondary sources. These included names of industry personnel, policy-makers and government officials; specialist industry terms such as projects and brands; and relevant business associates. Alternative spellings were also searched. The progressive growth and search capability of the BATDA collection,^35^ ^36^ in particular, permitted systematic review of BAT documents using recognised euphemisms for smuggling such as “transit”,^37^ “duty not paid”^38^ and “general trade” (GT).^39^ Advanced Boolean searches were undertaken using combined keywords. A total of 2340 documents were reviewed.

Documents were indexed in a specialist database to enable the construction of a historical and thematic narrative. Further analysis incorporated validation techniques within a hermeneutic process,^40^ including corroboration of interpretation between authors. Triangulation of findings was undertaken by supplementing industry documents with secondary sources—namely, newspaper articles and industry publications.

## RESULTS

### The growth of cigarette smuggling

“[T]o actively promote…[B&W] brands as far as the civil commotion situation permits”.^41^

Documents suggest cigarette smuggling in Lebanon has been a longstanding problem. An anti-smuggling squad within the Regie operated as early as the 1930s until 1976, then ceased during the civil war, before being reinstated in 1993.^17^ A 1954 BAT report describes “the Regie…somewhat worried about the considerable increase in smuggling. Only the day before, fifteen cases of “Lucky Strike” had been found on the beach near Beirut, the cases being marked ‘Tangier – in transit’ ”.^42^ The issue is also noted in three unpublished university theses on the Regie which cite smuggling routes via Gibraltar, Tangiers, Hong Kong, Limassol, Port Said and Anvers.^43^^–^45

It was from the early 1970s that smuggling grew to significant levels.^46^ ^47^ In large part, this was due to weakened government control over customs and excise during the civil war, coinciding with the plummeting of domestic production and legal imports by the Regie. As described by Philip Morris, “[r]ecent difficulties with the Lebanese Monopoly have lowered orders and resulted in quantity shortages in the normal imported market”.^48^ As legal supplies declined, “the transit [contraband] markets have filled the gap”.^48^ BAT reported that “[c]igarette production during the years of the civil war (which lasted 15–16 years) has come down to practically zero and [legal and illegal] imports into Lebanon rose to 13–15 billion cigarettes”.^49^

As cheap contraband flooded the market, it created fierce competition for dwindling supplies of local brands. Threatening strike action, Regie staff proved unsuccessful at securing government support for stronger regulation of import prices, as well as upgrading of manufacturing equipment.^50^ ^51^ While the media reported poor financial management by the Regie,^52^ former director-general Walid Salam saw the failure of the government to control smuggling as the real problem (interview with Walid Salam, former Regie director-general, Beirut, 22 May 2006). This is supported by documents that describe direct appeals by the Lebanese government to TTCs. In 1973, Phillipe Serhal (director-general Regie) asked Lorillard to “conform to the rules of business ethics”^53^ and cease supplying smugglers. He warned that the Regie would “reconsider our entire policy of the legal importation of your products for sale in our internal market”.^53^ In 1977 the Regie met with Lorillard again with the “stated…intention of helping stop transit sales”.^54^ It warned that “effective April 1977 the Lebanese authorities, with the backing of the Arab peace forces…were going to do their utmost to stop all irregular shipments to Lebanon, including jail and heavy penalty sentences for illegal importers”.^55^ Later the same year, a meeting was held in Paris with representatives of BAT, Philip Morris, RJ Reynolds (RJR), Rothman’s subsidiary Tobacco Exporter International, and Liggett and Myers. Khoury (director-general, Regie) complained that “50% of current sales in the Lebanon of imported brands were from G.T. [general trade] sources”.^56^ He requested TTCs not sell to distributors involved in contraband, and that company agents identify the sources of transit products.^56^ In 1978, Jacque Dagher (director-general, Regie) wrote to the president of BAT’s US subsidiary Brown & Williamson (B&W), copying to Cyprus-based distributor Peregrini (see below), the MOF, and the cultural attaché of the US embassy in Lebanon. The letter requested the company “stop all extra shipment to Cyprus, or any other country in our Region…with the intent to have them smuggled into Lebanon”.^57^

Despite these appeals, contraband continued to flood the Lebanese market.^58^ By 1983, “[l]egal imports account[ed] for only about a third of foreign cigarettes entering Lebanon”.^59^ Indeed, TTCs saw the civil war as an opportunity to expand sales in the country and region. B&W’s corporate strategy in 1982 stated that the company would “actively promote BWIT [B&W International Tobacco] brands as far as the civil commotion situation permits”.^41^ Its 1989–93 corporate plan described Lebanon as a growing market because “local manufacturing industries remain incapable of satisfying consumer demand vis-a-vis both quantity and quality”.^60^ Similarly, Philip Morris saw contraband as an opportunity to expand market share: “In most markets there are manufacturing capacity shortages necessitating some imports, legal or otherwise, if demand is to be quantitatively satisfied”.^61^ Philip Morris would “[m]aintain flexible pricing policy”^62^ given a “continued lack of security…to fully exploit the duty-free business and local products will be unable to regain a significant share of the market due to the continuation of low prices for imported products”.^63^

The government continued efforts to control smuggling, given its impact on Regie sales, which “slowed down due to the lower price of contraband Marlboro”^64^ and, in turn, tax revenues. One report described government measures “to avoid a complete collapse of the national economy”,^65^ which included “closure of the illegal ports”.^65^ In 1985, the Regie again “requested all manufacturers to stop DNP [duty not paid] shipments”.^66^ In February 1986, MOF Camille Chamoun complained to B&W about financial losses to the government, and the intention to put smugglers on trial and prohibit them from doing business in Lebanon.^67^ On 16 June 1986, a letter by the Ministry of Finance to Robert Gould (cultural attaché, US Embassy in Lebanon) complained of the illegal trade through Cyprus: “[I]f continued, [will] jeopardise many years of good relations and total business to American companies that amounts to 80 million dollars a year”.^68^ However, industry sources reported that “[m]ost observers judge that the Government has neither the political will nor the military strength” to effectively tackle the smuggling problem.^65^

The documents suggest these appeals had little effect on contraband sales.^69^ A 1984 BAT report stated that TTCs “are now treating transit business as a regular customer”.^70^ Indeed, the prospect of gaining a manufacturing presence in Lebanon was even seen as a potential threat to the profitable contraband trade. Nick Brookes (BAT, NBD) noted that “the Cyprus export business would obviously be impaired if we set up a business in Lebanon and import tariffs were raised”.^71^ Nick Gourlay (BAT, NBD) wrote to Keith Owen (BAT, Human Resources) noting that “[r]egarding the longer term, there are a number of different options for BAT to invest in Lebanon, each involving influential local interests. These options will need to be evaluated in the context of B&W’s substantial existing export business to the Lebanese monopoly (Regie)”.^72^

### The smuggling route to Lebanon: the role of transit agents

As elsewhere, TTCs worked through middlemen to supply contraband to Lebanon. A key player from the mid 1970s was Cyprus-based Kental Traders Limited, which received vast consignments from BAT and smuggled them to the Middle East and Africa. Cyprus has long been recognised as “a major contract manufacturer and transit supplier to countries such as Syria, Lebanon and Jordan”.^73^ Emil Schildt, BAT’s transit coordinator based in Hamburg,^29^ described Kental as

a very valuable customer for our [BAT] Group, who have extensive connections in transit in Middle East, what they proved during their 15 years agency for Lorillard and B&W. During the period 1976–1981 for instance, Kental sold in transit to Lebanon, Syria and Turkey more than 2 billion KENT.^74^

TA Macleod, of B&W Overseas based in Cyprus, claimed that “B&W through Kental, seized the opportunity of economic chaos to launch a portfolio of US brands at penetration price levels onto these markets”.^75^ Consequently, market share “expanded rapidly and within eight years reached a peak of 53.2% of total Levant imports (1993)”.^75^

The relationship between BAT and Kental is further revealed in documents concerning BAT’s relationship with Lebanese businessman, Souren Khanamirian. As a Regie board member and parliamentary member, Khanamirian was recruited by BAT to facilitate Kental’s operations in Lebanon. A report by the Center on Public Integrity described this relationship:

BAT had contracted with Kental to pay a Lebanese trader named Souren Khanamirian $2 for every case of cigarettes smuggled into Lebanon. At the time, Khanamirian was a board member of the Regie….Khanamirian complained in 1983 to BAT that he had not received his commission on the shipment. BAT settled the matter in March 1983 by paying the commission from its own accounts directly to Khanamirian. BAT then billed Kental for reimbursement. The BAT document notes that Khanamirian was paid a commission for “transit deliveries that go to Lebenon [sic].^28^

In 2001, Kental denied allegations of involvement.^76^ Eventually, Khanamirian’s services were terminated, with B&W publicly denying knowledge of his involvement in smuggling:

Brown & Williamson terminated its relationship with Khanamirian because of his failure to perform. B&W did not and does not condone the smuggling activities into and through Lebanon. The civil strife and economic situation in Lebanon are the cause of the lack of enforcement of customs law. It is the Lebanese government’s responsibility to ensure compliance with its customs laws.^77^

Another company that served as middleman was Cyprus-based Peregrini, a subsidiary of the Abela Group, a major Lebanese conglomerate. Peregrini served as a middleman for B&W, as revealed through a legal case brought in 1989 by Francois Mounayer, a former employee, for unfair dismissal.^78^ He alleged “smuggling into Lebanon, a hint of bribery, and unlawful trading with Iran during the American hostage crisis”.^79^ He stated that “B&W’s intent was to sell its cigarettes to Abela’s subsidiary which would, in turn, illegally smuggle them into Lebanon”.^80^ While the Regie had monopoly control over imports, B&W documents state that “Peregrini has two major customers for Kent de Luxe—the Lebanese Army and Mr Tbeily, a gentleman of Jewish faith who resides in the Lebanon”.^81^ The relationship between Mounayer and B&W soured over “parallel imports”, a term used by the industry to describe onward smuggling by middlemen to markets unauthorised by the TTC. It was alleged that “B&W was worried that if Mounayer was independently engaged in illegal sales to other countries that it would somehow jeopardize B&W’s position in the Middle East and result in business losses”.^80^ In 1983 B&W vice president Tom Whitehair wrote to Abela requesting that “Mounayer be taken off our business completely”.^80^ In court, B&W denied the claims as “totally without merit”,^77^ pointing to Mounayer’s own admission that he was “willingly engaged in illegal cigarette smuggling”.^80^ B&W official response stated:

The nature of the cigarette business is such that Brown & Williamson sells to a range of independent international wholesalers and distributors. Where, when and to whom they sell the product is a matter for the wholesaler concerned. Brown and Williamson does not smuggle cigarettes … . The control of this trade is ultimately the responsibility of the governments concerned. Brown & Williamson has on numerous occasions, either directly or through industry groups, urged local governments to take effective action to prevent this trade since it damages Brown & Williamson’s efforts to lawfully conduct business in these countries.^77^

In 1990 B&W won the case “at minimal cost and without any discovery”.^82^

### A conduit for supplying neighbouring countries

As well as a target market, Lebanon was a key “vehicle for distribution” in the Middle East and Africa.^83^ The Levant region (Lebanon, Cyprus, Syria and Jordan) was described as an “intertwined group of markets with very fluid and changing channels of distribution”.^84^ During the 1980s, “[o]ver half of all imports [into Lebanon], legal and illegal, are then further smuggled out to Syria, Jordan, Iraq and Turkey”.^59^ Kent and Marlboro products smuggled through Lebanon were also found in Israel.^85^

The most important among these secondary markets was Syria,^86^ which, given an import ban in 1981,^87^ was only accessible to TTCs through smuggling. With “porous borders between the two countries”,^88^ Lebanon was “an important conduit cigarette market to Syria with additional volumes of approximately 6 bns p.a being reexported [smuggled] to Syria mainly because import duties in Lebanon are currently very low”.^89^ Consequently, “[v]irtually all imports were done by the Regie, DNP and 5–6 bn would stay in Lebanon and 9–10 bn go across the border into Syria”.^90^ In 1988 JK Booth (BATUKE) requested Youseff Abdallah to survey BAT brand availability following their supply, via the contraband market, to Syria: “As you know, we recently supplied a company called WORLDWIDE with Benson & Hedges Special Filter for end destination Syria”.^91^ Abdallah reported back:

Many private ports feed freely the Syrian transit points, but, basically through the Lebanese border town of Shetoura, which is 10 miles from the Syrian borders. The reason for the importance of Shetoura as a main transit point into Syria, is the fact the military presence of Syria starts from Shetoura and from there into Lebon up to Beirut. We must always remember that the Army is enjoying great influence and they are the spear head of the transit Mafia. From Shetoura stocks are bought and sent across a number of points over the hills in Mules and Jeep vehicles—Into the Syrian border twons [sic], stocks are hidden in many different under-ground stores, and only small quantities are displayed in the wholesalers shops.^92^

Similarly, the Philip Morris board of directors described extensive smuggling to Syria via Lebanon: “[Philip Morris] dominate Lebanon with an overall market share of 65% stressing however that although there are no direct [legal] sales to Syria and Jordan the overflow [contraband] from Lebanon generates estimated shares in these two markets of 18% and 9%, respectively, again making us the leaders of the international segment”.^61^

BAT continued “to supply Kental in Cyprus with UK manufactured stock … into Lebanon/Syria as transit”^93^ throughout the 1980s “to respond to immediate opportunities in what will continue to be a volatile environment”.^94^ As Syrian troops entered Lebanon, contraband flows increased:

The arrival of Syrian troops in West Beirut has resulted in a loosening of the Syrian border, and fairly substantial quantities are moving into the country via the Bekaa valley.…and Kental has placed a series of orders to capitalize quickly on this opportunity.^95^

New or substitute supply routes were established when needed:

During the last quarter of 1986 the main route into Syria via the Bekaa closed. As a result shipments were recovered via Tripoli, thus allowing us to maintain access but at reduced volumes. In early February the route via the Bekaa reopened and is expected to remain open at least for the next 3 to 6 months. With the arrival of Syrian troops in W. Beirut it is likely that goods will now move even more freely.^96^

Indeed, Abdallah’s report of the Syrian market describes how troops were a key part of the supply chain from Lebanon to Syria and onwards:

An official transit operation-encouraged, handled and financed by the Army troops, the Pole [Police] Force and the Security Force—Every one gets a share of the profit’s cake. The transit business is mainly an American Blend Market, and UK brands are limited and their volume is mainly going to Jordan via Syrian border points with Jordan.^93^

This corresponded with earlier reports by BAT that smuggling “in great Beirut [is] (under the control of the Lebanese army aided by the Multi National Force)”.^97^

### Reliance on legal and illegal trade: “dual distribution channels into key markets”

By the late 1980s, three factors affected the contraband trade, which led TTCs to reflect on their strategic use to facilitate market access. First, the Lebanese government sought to take over the trade itself. Given ineffectual appeals for TTCs, and an inability to control border crossings and sales outlets “due to the political situation and foreign occupation”,^98^ the government announced in October 1987 that “Lebanese transit ports” would be “officially” closed. Under strict rules^99^ ^100^ “the monopoly will take full control of cigarette imports, thereby closing previous routes for the importation of duty free [smuggled] cigarettes”.^101^ The Regie “made arrangements with all ‘de facto’ zones from the north to the south of Lebanon to ensure proper distribution”.^101^ Local newspapers reported the Regie contracted several private companies affiliated with, or having some connection to, various local militia groups.^102^ These companies were paid 8% of Regie profits,^103^ and all smuggled cigarettes were to be confiscated by them.^104^

This arrangement was solidified under Regie director-general Salam who, upon assuming office in 1991, reached agreement with the companies, agents and distributors smuggling through Cyprus to route the products through the Regie. According to Salam, “Instead of 50,000 [cases] sold through me and 50,000 of smuggled products…now I got sold through me in the Regie 100,000 cases….We put down the smuggling and turnover through me increased” (Interview with Walid Salam, 22 May 2006). Given direct supply to the Regie,^105^ Kental was “no longer as influential as it was”^106^ although it continued to collect commission fees as B&W saw “[t]heir future use…in Syria where they have good contacts”.^107^ Correspondingly, onward smuggling to neighbouring countries continued:

We sold it in Lebanon (like Marlboro) and others took it outside on their own responsibility. It was their own problem. They took it to Syria, Iran and Iraq. We did not do anything illegal since we were not the ones smuggling, we are selling them here … but the real market in Lebanon is between 50 to 55,000 cases…we reached up to 120,000 cases a month. (Interview with Walid Salam, 22 May 2006.)

TTCs anticipated the Regie’s attempts at controlling smuggling “to be limited and short-lived”,^60^ and the strategy was criticised by the local media.^108^ None the less, by 1991 the combined Lebanese-Syrian market reached “20 bns [sticks] per annum, 13 bns believed to be imported via Cyprus. BWIT held 54% in 1989, PMI 28% and RJR 17%”.^109^

A second factor affecting TTC strategy was the problem of controlling the activities of middlemen. The ongoing problem of “parallel imports” by distributors, tempted by lucrative profits, at times resulted in oversupply: “Parallel shipments have been controlled over the quarter as far as possible, and in recent weeks we have ceased shipments to Cyprus as a result of an overstock situation arising in the Lebanon”.^110^ In a 1988 job description for a BATUKE Red Sea/central area manager, the incumbent’s role was

To evaluate routes, prices and risks, then to recommend action…to exercise judgement over sales/shipments when markets are supplied by more than one source. Constant vigilance is necessary in pricing because of the competitiveness of the area and the problem of parallel shipments between various territories within the Middle East and West Africa.^111^

The consequent downward pressure on prices threatened to harm both local brands and legal imports. The Regie responded by lowering the price of its brands so that “it is not worth the amount of profit they got through smuggling” (Interview with Walid Salam, 22 May 2006).

Third, the end of civil war in Lebanon in 1991 led to the possibility that the Regie would revive local production and legal imports. In 1993, Mike Baker (BAT, regional export manager Middle East)^112^ observed that Cyprus/Levant remained the “centre of GT business for the southern and eastern Mediterranean”,^113^ but anticipated that “within 2 years the authorities in Lebanon will make substantial progress to legitimise the tobacco industry”.^114^ BAT reported disruption that year to some smuggling routes which led to decreased sales.^114^ Documents describe “a shift from current [illicit] product sourcing” alongside a “more stable and…highly competitive” Lebanese market.^115^ B&W thus predicted “a continuing trend to duty paid business in Syria and Lebanon”.^116^

Within this context, TTCs had a close interest in how the Regie’s monopoly status might change. As previously, the potential benefits of an agreement between BAT and the Regie were carefully weighed against the risks to the contraband trade. Of particular importance was Lebanon’s role within a “broader strategy for the Middle East, or at least Lebanon-Syria-Jordan”.^116^ B&W anticipated “a possibility that the Syrian market will open to direct cigarette imports during 1992”.^117^ This was eventually achieved in 1993 as the government sought to combat smuggling to “gain additional customs and duties revenues of USD 60 mn”.^118^ B&W remained apprehensive that “[a]ny significant investment in Syria that increased domestic capacity would be likely to disrupt the existing flow of product from Lebanon to Syria”.^119^ Thus, BAT sought an arrangement with the Syrian Regie similar to that in Lebanon:

The Syrian market is approx. 13.5 bns for which the Syrian Regie has capacity for only about half. The remainder is imported and for which Lebanon acts as an important conduit. The Lebanese Regie imports the product, and sells it to wholesalers who then transit it to Syria. It would be in the best interests of the Lebanese and Syrian Regies as well as the foreign manufacturers if the business was dealt with between the two Regies, cutting out the middle men. The Syrian Regie could pay the Lebanese in Syrian pounds. Syria represents a big opportunity and the Lebanon may hold the key to a combined market whose total volume exceeds 19 bns per year.^120^

Concerns about competition from two revived Regies, however, have proved premature given ongoing instability in the region. Despite the end of the war, B&W noted that the conflict was “unlikely to be resolved in the near future”.^121^ This prediction proved true, in turn impacting on cigarette imports which declined by 60% between 1995 and 2002.^122^ The company maintained “dual [legal and illegal] distribution channels into key markets”.^118^ It was concluded that “[b]ecause a number of markets cannot meet demand (i.e. Lebanon, Syria, Afghanistan), parallel shipments are filling the voids”.^123^ In 2006, extensive smuggling still existed in the region.^124^

## DISCUSSION

This review of industry documents reveals once again how the contraband trade has been integral to TTC strategies to expand market access in emerging markets. However, while cigarette smuggling has been a global problem, Lebanon demonstrates the particular challenges faced by countries with weak governance. The domestic tobacco industry’s entanglement with particular political factions has meant that policy has been driven by economic, rather than public health, concerns. This is illustrated by the government’s indecision over the Regie’s legal status, as well as involvement of groups linked to militia groups to take over the contraband trade in order to regain revenues, a general practice that has kept the government weak to the present day.^125^

Within this context, TTCs saw weak governance as an opportunity to further its market access strategy. Resisting appeals to cease supplying contraband to the country contributed to the government’s revenue problems by undermining Regie sales and circumventing import duties. TTCs recognised the weak position of the government to address the smuggling problem, seeking to use it as leverage to negotiate a manufacturing presence in Lebanon. As well as circumventing the Regie’s monopoly control of imports, contraband was channelled through Lebanon to reach the closed markets of Syria, Jordan and other neighbouring countries. Initially a means of selling more cigarettes and building market share, often through “penetration price levels”,^75^ smuggling became an important part of broader objectives to secure a manufacturing presence in the country. To date this has not been achieved because of ongoing uncertainty about the Regie’s monopoly status amid political instability.

The findings of this paper support the need for concerted attention to cigarette smuggling in the Middle East through a dedicated protocol of the FCTC. The crossborder nature of the smuggling problem, confirmed by this analysis, requires collective action across the region. Such a protocol should cover tracing and tracking systems, obligations on TTCs to control their supply chain, enforcement and prosecution mechanisms, anti-money laundering measures, record keeping of imports and exports, and sharing of information across countries.^126^

Importantly, this paper shows that the tobacco industry should not participate in the negotiation of such a protocol, and extreme caution should be exercised towards recent industry overtures that appear to support stronger measures to tackle smuggling. In 2007 Philip Morris and BAT signed memoranda of understanding with the Lebanese MOF on anti-smuggling activities as part of their corporate social responsibility initiatives.^127^ ^128^ Similarly, JTI have conducted anti-smuggling events in cooperation with the Regie and customs officials.^129^ On 23 February 2008, Philip Morris organised a training session for the Regie smuggling squad as part of the activities in the agreed signed memorandum.^130^ Notably, such efforts have disingenuously focused on the separate issue of counterfeits, which compete with the legal and illegal sale of genuine products, and on “incentives and motivations” for smuggling notably high tobacco taxation.^131^ While TTCs have continued to publicly deny involvement in cigarette smuggling, the evidence presented here adds to previous analyses of the complicity of the tobacco industry in the contraband trade.

## CONCLUSION

Cigarette smuggling, comprising around one-quarter of exports,^132^ undermines public health by making cheaper cigarettes more readily available and, in turn, encouraging increased consumption.^133^ The tobacco industry benefits from gaining access to closed or regulated markets, increasing revenues,^134^ and discouraging governments from raising tobacco taxes.^135^ Countries such as Lebanon, which face chronic political and economic instability, are particularly vulnerable to such activities. Collective action is needed, both regionally and globally, to achieve a designated FCTC protocol to effectively tackle this global problem, as well as increased support to countries such as Lebanon in implementing its measures.

**What this paper adds**

Previous research of European, Asian, North American and Latin American countries documents the complicity of TTCs in cigarette smuggling. To date there has been limited analysis of the Middle East owing to the lack of available documents. With BAT documents from the Guildford and Minnesota depositories now online, and drawing on secondary data from local sources, this paper provides a detailed account of Lebanon as one of the most strategically important countries in the region to TTCs for expanding market access.

## References

[1] Assaad R Roudi-Fahimi F Youth in the Middle East and North Africa: demographic opportunity or challenge? Population Reference Bureau, 2007

[2] UN Population Fund State of world population 2007. New York: United Nations, 2007

[3] Special Report: Tobacco industry, Sparking up. Executive (Beirut) November 2007:38–42

[4] Middle East Market Analysis MENA market review. *World Tobacco* May 2005

[5] World Bank Tobacco: health impacts and economics in the Middle East and North Africa Region. Washington: Human Development, Middle East and North Africa Region, 2002

[6] Jha P Kent R Son N Estimates of global and regional smoking prevalence in 1995, by Age and Sex. Am J Public Health 2002;92:1002–6 12036796 10.2105/ajph.92.6.1002 PMC1447501

[7] Saade G Abou Jaoude S Afifi Soweid R Patterns of tobacco use: results from GYTS in Lebanon. WHO Eastern Mediterranean Health Journal 2008;(in press). 19161103

[8] Draft law may reduce cigarette consumption. Daily Star Newspaper 2004; http://www.tobacco.org/news/168595.html (Accessed 4 April 2005)

[9] Shechter R Multinational interference and its demise. Smoking, culture and economy in the Middle East, The Egyptian Tobacco Market 1850–2000. London: IBTauris, 2006:96–116

[10] Cox H The growth of an international cigarette industry. The global cigarette origins and evolution of British American Tobacco 1980–1945. Oxford: Oxford University Press, 2000:3–16

[11] Report on tobacco companies in their respective countries. 13 March 1961. British American Tobacco. http://bat.library.ucsf.edu/data/h/j/h/hjh36a99/hjh36a99.pdf

[12] Regie Libanaise des Tabacs et Tombacs Official website. www.regielibanaisedestabacs.com (Accessed 3 May 2007).

[13] Allanby J Letter regarding circular from Lebanese Government, 2 Aug 1961. British American Tobacco. http://bat.library.ucsf.edu/data/b/n/k/bnk40a99/bnk40a99.pdf

[14] Regie workers’ syndicate meets the Prime Minister. *Al Safir Daily Newspaper* 16 May 1974

[15] Ministerial dispute regarding the Regie. *Al Safir Daily Newspaper* 15 July 1974

[16] Urgent meeting to discuss Regie issues: reserve workers rights and postpone discussion about its status. *Al Safir Daily Newspaper* 14 July 1974

[17] Team International Restructuring the Regie. *Interim audit report, Investment Development Authority.* Beirut: Team International, Idosuez Capital, E. Kanaan & Co, 1996

[18] Shafey O Dolwick S Guindon G E, eds. Tobacco control country profiles. Atlanta: American Cancer Society, 2003

[19] MacKay J Eriksen M Shafey O The tobacco atlas. 2nd ed. Washington, DC: American Cancer Society, 2006

[20] British American Tobacco Official corporate website. How we tackle illicit trade; http://www.bat.com/oneweb/sites/uk__3mnfen.nsf/vwPagesWebLive/C17D2C9CCC29CB27C12571EA0058EAF9?opendocument&SID = &DTC = (accessed 2 May 2007)

[21] Framework Convention Alliance How big was the global illicit tobacco trade problem in 2006? June 2007. http://www.fctc.org/x/documents/HowBigWasTheIllicitTobaccoTradeProblem_2006_English.pdf (accessed 2 May 2007)

[22] Gilmore AB McKee M Moving east: how the transnational tobacco companies gained entry to the emerging markets of the former Soviet Union. Part I: Establishing cigarette imports. Tob Control 2004;13:143–50 15175531 10.1136/tc.2003.005108 PMC1747849

[23] Joosens L Raw M Cigarette smuggling in Europe: who really benefits? Tob Control 1998;7:66–71 10.1136/tc.7.1.66 PMC1759658 9706757

[24] International Consortium of Investigative Journalists Tobacco companies linked to criminal organizations in cigarette smuggling, China. Washington, DC: Center for Public Integrity, 2001 http://www.publicintegrity.org/report.aspx?aid = 352&sid = 100

[25] Collin J LeGresley E MacKenzie R Complicity in contraband: British American Tobacco and cigarette smuggling in Asia. Tob Control 2004;13 supp II:ii96–ii111 15564212 10.1136/tc.2004.009357 PMC1766170

[26] Lee K Collin J ‘Key to the future’: British American Tobacco and cigarette smuggling in China. PLoS Medicine 2006;3:228–37 10.1371/journal.pmed.0030228 PMC1502159 16834455

[27] Tobacco Free Kids The Big Cigarette Companies and Cigarette Smuggling. Washington, DC: National Center for Tobacco-Free Kids, 18 May 2003. http://tobaccofreekids.org/research/factsheets/pdf/0044.pdf (accessed 2 May 2007)

[28] International Consortium of Investigative Journalists Tobacco companies linked to criminal organizations in cigarette smuggling, Latin America. Washington, DC: Center for Public Integrity, 2001 http://www.publicintegrity.org/report.aspx?aid = 35 (accessed 5 May 2007)

[29] WHO EMRO The cigarette “transit” road to the Islamic Republic of Iran and Iraq: Illicit tobacco trade in the Middle East. Cairo: WHO Eastern Mediterranean Regional Office, 2003. http://www.emro.who.int/tfi/TFIiraniraq.pdf (accessed 2 May 2007)

[30] WHO EMRO Coveting Iran: The infiltration and exploitation of Iran by global cigarette companies. Cairo: WHO Eastern Mediterranean Regional Office, 2000. http://www.emro.who.int/TFI/IranReport.doc (accessed 8 Mar 2005)

[31] MacKenzie R Collin J Lee K The tobacco industry documents: an introductory handbook and resource guide for researchers. London: London School of Hygiene and Tropical Medicine, 2003

[32] Bero L Implications of the tobacco industry documents for public health and policy. Ann Rev Public Health 2003;24:267–88 12415145 10.1146/annurev.publhealth.24.100901.140813

[33] Balbach E Tobacco industry documents: comparing the Minnesota Depository and internet access. Tob Control 2002;11:68–72 11891371 10.1136/tc.11.1.68 PMC1747640

[34] Muggli ME LeGresley EM Hurt RD Big tobacco is watching: British American Tobacco’s surveillance and information concealment at the Guildford Depository. Lancet 2004;363:1812–9 15172782 10.1016/S0140-6736(04)16309-5

[35] Collin J Lee K Gilmore A Unlocking the corporate documents of British American Tobacco: an invaluable global resource needs radically improved access. Lancet 2004;363:1746–7 15172767 10.1016/S0140-6736(04)16334-4

[36] Lee K Gilmore A Collin J Looking inside the tobacco industry: revealing insights from the Guildford Depository. Addiction 2004;99:394–7 15049733 10.1111/j.1360-0443.2004.00718.x

[37] George-Perutz A Re: Transit Study. British American Tobacco. 25 August 1989. http://bat.library.ucsf.edu/data/q/h/m/qhm97a99/qhm97a99.pdf

[38] British American Tobacco Venezuelan Market Definitions and Assumptions. 25 August 1989. http://bat.library.ucsf.edu/data/r/q/m/rqm87a99/rqm87a99.pdf

[39] British American Tobacco Review of Asia-Pacific Market, January 1995. http://bat.library.ucsf.edu/data/l/y/b/lyb96a99/lyb96a99.pdf

[40] Forster N The analysis of company documentation. Cassell C Symon G, eds. Qualitative methods in organizational research: a practical guide. London: Sage, 1994:147–66

[41] Brown & Williamson Marketing and operational plans 1982. 10 Nov 1981. http://legacy.library.ucsf.edu/cgi/getdoc?tid = ibm33f00&fmt = pdf&ref = results

[42] Lebanese Regie 30 Oct 1956. British American Tobacco. http://bat.library.ucsf.edu/data/e/a/c/eac01a99/eac01a99.pdf

[43] Karyergatian V Monopoly in the Lebanese Tobacco Industry [Masters Thesis]. American University of Beirut, 1965

[44] Itr N The market of light cigarettes with special reference to Silk Cut [Masters Thesis]. American University of Beirut, 1984

[45] Salih M The Lebanese Regie of Tobaccos and Tambacs:current problems and recommended solutions [Masters]. Beirut: American University of Beirut, 1990

[46] Tug of war between Regie and smugglers: products are available and anti-smuggling squads are absent. *Al Safir Daily Newspaper* 25 Jul 1977

[47] Meeting with Rofayil regarding smuggling of cigarettes and how to curb. *Al Safir Daily Newspaper* 31 Aug 1977

[48] Philip Morris Five year plan 1976–1980. 1976. http://legacy.library.ucsf.edu/tid/kmp14e00/pdf

[49] Van Waay A Lebanon. 25 Jan 1994. British American Tobacco. http://bat.library.ucsf.edu/data/q/n/s/qns37a99/qns37a99.pdf

[50] Delaying Production of local Cigarettes serves foreign companies and their agents and causes great losses to the Regie. *Al Safir Daily Newspaper* 8 Jul 1974

[51] Minister of Finance discusses today the Regie Status and protection of local production. *Al Safir Daily Newspaper* 16 Jan 1975

[52] Heads of sale request from Karami and Chamoun to stop bribes and stealing in Regie. *Al Safir Daily Newspaper* 9 Jun 1985

[53] Serhal P Importation of cigarettes to Lebanon. 8 Jan 1973. *Lorillard*. http://ltdlimages.library.ucsf.edu/imagesq/q/f/r/qfr90e00/Sqfr90e00.pdf

[54] Richardson P Lebanon Leaf. 14 Nov 1977. British American Tobacco. http://bat.library.ucsf.edu/data/p/s/p/psp41a99/psp41a99.pdf

[55] Dominguez J Régie Lebanon. 28 Feb 1977. British American Tobacco. http://bat.library.ucsf.edu/data/k/z/p/kzp41a99/kzp41a99.pdf

[56] Drake RL Notes of Meeting called by the Lebanese Regie and held at SEITA Head Office, Paris. 4 Nov 1977. British American Tobacco. http://bat.library.ucsf.edu/data/q/s/p/qsp41a99/qsp41a99.pdf

[57] Dagher J [Letter to RM Kink]. 8 February 1978. British American Tobacco. http://bat.library.ucsf.edu/data/j/z/p/jzp41a99/jzp41a99.pdf

[58] Regie status unresolved for 20 years: importers flood the market with foreign cigarettes and local sales down leading to government losses. *Sawt Al Ahrar Daily* 11 September 1980

[59] Tobacco Merchants Association International Tobacco Report Tobacco Trade Barometer Part 7 B. US Tobacco Trade by World Region. 20 Sep 1983. *Lorillard*. http://legacy.library.ucsf.edu/tid/nlw74c00/pdf?search = %22nlw74c00%22

[60] Brown & Williamson 1989–1993 Corporate plan. 1988. http://legacy.library.ucsf.edu/cgi/getdoc?tid = npl33f00&fmt = pdf&ref = results

[61] Philip Morris EEMA Region presentation to the board of directors. Sept 1983. http://legacy.library.ucsf.edu/cgi/getdoc?tid = byt24e00&fmt = pdf&ref = results

[62] Philip Morris Five year plan 1984–1988. March 1984. http://legacy.library.ucsf.edu/cgi/getdoc?tid = hhk85e00&fmt = pdf&ref = results

[63] Philip Morris Lebanon Area V, 5-Year Plan. 1981. http://legacy.library.ucsf.edu/cgi/getdoc?tid = fhy19e00&fmt = pdf&ref = results

[64] Thoma W EEMA weekly highlights. 19–23 Mar 1984. Philip Morris. http://legacy.library.ucsf.edu/cgi/getdoc?tid = tpt24e00&fmt = pdf&ref = results

[65] Thoma W EEMA weekly highlights. 29 Oct-2 Nov 1984. Philip Morris. http://legacy.library.ucsf.edu/cgi/getdoc?tid = ept24e00&fmt = pdf&ref = results

[66] Telling W Bi-Monthly Update. 6 May 1985. Brown & Williamson. http://legacy.library.ucsf.edu/cgi/getdoc?tid = pww21f00&fmt = pdf&ref = results

[67] Chamoun C [Smuggling issue in Lebanon]. 21 Feb 1986. British American Tobacco. Bates No: 301183233. Available from Guildford Depository (accessed 15 June 2004).

[68] Chebil K [Letter by the Lebanese Ministry of Finance]. 16 Jun 1986. British American Tobacco. Bates No: 301183230/3232. Available from Guildford Depository (accessed 15 June 2004).

[69] Regie administration responds to the accusation: Tobacco Mafia. *Al Sharq* 18 April 1985

[70] Williams A [ATC’s decision to appoint Gallaher International].1984. American Tobacco Company. http://legacy.library.ucsf.edu/tid/hcx24f00

[71] Brookes N Lebanon. 31 Jan 1994. British American Tobacco. http://bat.library.ucsf.edu/data/k/n/s/kns37a99/kns37a99.pdf

[72] Gourlay N Re: Project Leader for Lebanon/Jordan. 14 Jul 1994. British American Tobacco. http://bat.library.ucsf.edu/data/k/m/s/kms37a99/kms37a99.pdf

[73] Gourlay N B&W Margins in the Mideast/Africa. 25 Apr 1988. British American Tobacco. http://bat.library.ucsf.edu/data/v/l/t/vlt01a99/vlt01a99.pdf

[74] Schildt E British American Tobacco.1983. Commission payment to S. Khanamirian. British American Tobacco. Bates No: 301515421/54222. Available from Guildford Depository (accessed 15 June 2004).

[75] Macleod TA Re: Lebanon-Draft Strategy Document. 16 Aug 1995. British American Tobacco. http://bat.library.ucsf.edu/data/p/k/s/pks37a99/pks37a99.pdf

[76] Matthew J Company denies cigarette smuggling allegations. Cyprus Mail 2001. http://www.hri.org/news/cyprus/cmnews/2001/01-12-18.cmnews.html#01

[77] Brown & Williamson Position statement 23 Apr 1990. http://bat.library.ucsf.edu/data/t/u/u/tuu34a99/tuu34a99.pdf

[78] US District Court United States District Court for the Southern district of New York. [1989]. British America Tobacco. http://bat.library.ucsf.edu/tid/cbd34a99

[79] Frigon H [Letter to P Sheehy]. 30 Aug 1989. British American Tobacco. http://bat.library.ucsf.edu/data/m/g/q/mgq54a99/mgq54a99.pdf

[80] Hendershot ML [Letter to Nick Cannar]. 15 Oct 1990. Brown & Williamson. http://bat.library.ucsf.edu/data/x/u/k/xuk50a99/xuk50a99.pdf

[81] Investigation on transit shipments. 1 Jun 1987. British American Tobacco. http://bat.library.ucsf.edu/data/a/v/k/avk50a99/avk50a99.pdf

[82] Corporate long-term performance objectives. 1990. British American Tobacco. http://bat.library.ucsf.edu/data/o/f/w/ofw00a99/ofw00a99.pdf

[83] BAT 1988–1992 five year plan. Jan 1998. British American Tobacco. http://bat.library.ucsf.edu/data/r/t/o/rto20a99/rto20a99.pdf

[84] Sandefur T [Letter to UG Herter]. 14 Sep 1993. British American Tobacco. http://bat.library.ucsf.edu/data/o/o/j/ooj11a99/ooj11a99.pdf

[85] Stephenson R Israeli production can double if market opens. Dec 1979. Brown & Williamson. http://legacy.library.ucsf.edu/cgi/getdoc?tid = phk90f00&fmt = pdf&ref = results

[86] 1.3 million box to Syria in 1991: stopping smuggling did not work, 3 million into Lebanon and beyond. *An Nahar Daily* 1 Jun 1992

[87] Syrian crop reduction due for 1982; smuggled foreign brands abound. Tob Int 1982;6:5–8

[88] Aitken D Jordan and Lebanon meeting notes. 28 Oct 1994. British American Tobacco. http://bat.library.ucsf.edu/data/z/l/s/zls37a99/zls37a99.pdf

[89] O’Hara S Syria. 14 Dec 1994. British American Tobacco. Bates No: 503859032/9034. *Available from Guildford Depository* (Accessed 15 June 2004).

[90] Van Waay A Lebanon. 25 Jan 1994. British American Tobacco. http://bat.library.ucsf.edu/data/q/n/s/qns37a99/qns37a99.pdf

[91] Booth JK Syria market visit. 19 September 1988. British American Tobacco. http://bat.library.ucsf.edu/data/f/v/g/fvg38a99/fvg38a99.pdf

[92] Abdalla Y Trip notes—Syria-21st-28th Sept, 88. 3 October 1988. British American Tobacco. http://bat.library.ucsf.edu/data/j/v/g/jvg38a99/jvg38a99.pdf

[93] Reynolds G Subject: Lebanon. 1987. British American Tobacco. Bates No: 301554795. Available from Guildford Depository (accessed 6 June 2007).

[94] Wisner J Monthly Report, May 1986. 23 Jun 1986. Brown & Williamson. http://legacy.library.ucsf.edu/cgi/getdoc?tid = eta30f00&fmt = pdf&ref = results

[95] Wisner J Monthly report. Feb 1987. Brown & Williamson. http://legacy.library.ucsf.edu/cgi/getdoc?tid = eaj61f00&fmt = pdf&ref = results

[96] Miningham G Re: Lebanon/Cyprus—trip notes February 17–21, 1987. 24 Feb 1987. Brown and Williamson. http://ltdlftd.library.ucsf.edu/action/document/view?tid = jne61f00

[97] Staunskjaer B Lebanon trip notes17 to 20 March 1983. 20 Mar 1983. British American Tobacco. Bates No: 301515409/5410. Available from Guildford Depository (accessed 15 June 2004).

[98] Brown & Williamson Corporate plan 1992–1996. 1992. British American Tobacco. http://bat.library.ucsf.edu/data/c/v/i/cvi31a99/cvi31a99.pdf

[99] Regie decides to fight smuggling. *Al Safir Daily* 22 Sep 1987

[100] Wisner J Monthly Report, September 1986. 21 Oct 1986. Brown & Williamson. http://legacy.library.ucsf.edu/cgi/getdoc?tid = cta30f00&fmt = pdf&ref = results

[101] Henchley R Legal Matters—October board meeting.1 Oct 1987. British American Tobacco. http://bat.library.ucsf.edu/data/h/q/w/hqw10a99/hqw10a99.pdf

[102] Anti smuggling given to private company. *Al Safir Daily* 25 Oct 1987

[103] Militia companies given the task to stop smuggling by sea, air, and land. *Al Safir Daily* 24 Dec 1987

[104] Regie director explains how private companies are fighting smuggling: smuggling almost stopped. Al Anwar Daily 1987;5 Oct.

[105] BATUKE Company plan 1992–1996. Oct 1991. British American Tobacco. http://bat.library.ucsf.edu/data/j/d/p/jdp70a99/jdp70a99.pdf

[106] Green JL Meeting between BWIT and BATUKE on Friday 19 July 1991. 22 Jul 1991. British American Tobacco. http://bat.library.ucsf.edu/data/u/k/w/ukw31a99/ukw31a99.pdf

[107] Tweed H Lebanon. 1994. British American Tobacco. http://bat.library.ucsf.edu/data/u/l/s/uls37a99/uls37a99.pdf

[108] Nine year old procedure: decision to allow militias to sell Regie products. *Al Safir Daily* 27 Sep 1987

[109] BATUKE Company plan 1991–1995, Part I. October 1990. British American Tobacco. http://bat.library.ucsf.edu/data/i/s/i/isi87a99/isi87a99.pdf

[110] 1st Quarter PAO Report. 1981. Brown & Williamson. http://legacy.library.ucsf.edu/cgi/getdoc?tid = wye33f00&fmt = pdf&ref = results

[111] Booth K Job description—area manager—Red Sea/Central.13 Nov 1987. British American Tobacco. http://bat.library.ucsf.edu/data/s/u/w/suw95a99/suw95a99.pdf

[112] Clarke PL Organisation. 15 Oct 1993. British American Tobacco. http://bat.library.ucsf.edu/data/o/m/k/omk41a99/omk41a99.pdf

[113] Baker M Notes on ME trip. 1993. British American Tobacco. Bates No: 301661793/1795. Available from Guildford Depository (Accessed 15 June 2004).

[114] Souza Cruz Major brands performance. 1993. British American Tobacco. http://bat.library.ucsf.edu/data/b/u/k/buk80a99/buk80a99.pdf

[115] Brown & Williamson 1994–1998 five year plan. Dec 1993. British American Tobacco. http://bat.library.ucsf.edu/data/j/c/l/jcl11a99/jcl11a99.pdf

[116] Van Waay A Lebanon. 1994. British American Tobacco. http://bat.library.ucsf.edu/data/f/n/s/fns37a99/fns37a99.pdf

[117] Brown & Williamson 1992–1996 corporate plan. Jan 1992. British American Tobacco. http://bat.library.ucsf.edu/data/j/r/o/jro90a99/jro90a99.pdf

[118] British American Tobacco Lebanon: Barclay launch 1995. Bates No: 500331198. Available from Guildford Depository (accessed 15 June 2004).

[119] Gourlay N Lebanon. 8 Mar 1995. British American Tobacco. http://bat.library.ucsf.edu/data/h/l/s/hls37a99/hls37a99.pdf

[120] Tweed H Lebanon. 28 Sept 1994. British American Tobacco. http://bat.library.ucsf.edu/data/d/m/s/dms37a99/dms37a99.pdf

[121] Brown & Williamson 1991–1995 corporate plan. Jan 1991. British American Tobacco. http://bat.library.ucsf.edu/data/r/g/s/rgs51a99/rgs51a99.pdf

[122] Regie Total Import Units of Cigarettes. http://www.regielibanaisedestabacs.com/images/graph4.jpg (accessed 14 January 2008)

[123] Brown & Williamson Brown & Williamson Tobacco Corporation. Jan 1991. British American Tobacco. http://bat.library.ucsf.edu/data/r/g/s/rgs51a99/rgs51a99.pdf

[124] Zawya Middle East Business News and Company Directory Smoke smuggling. Executive. November 2006. http://www.zawya.com/story.cfm/sidZAWYA20061107122448 (accessed 5 January 2006)

[125] Adwan C Corruption in reconstruction: the cost of national consensus in post-war Lebanon. Center for International Private Enterprise 1 Dec 2004. http://www.cipe.org/pdf/publications/fs/adwan.pdf (accessed 20 Mar 2007)

[126] Campaign for Tobacco Free Kids Illicit trade: questions and answers. http://tobaccofreecenter.org/files/pdfs/ILL-%20QA-jan08.pdf (accessed 23 Feb 2008)

[127] Philip Morris to help curb cigarette smuggling in Lebanon. *Daily Star* Sept 2007

[128] BAT signs memorandum of understanding. *An Nahar Daily* 1 Nov 2007

[129] Training campaign to fight smuggling and counterfeit cigarettes. *An Nahar Daily* 8 Nov 2007

[130] Training session in cooperation between the Regie and Philip Morris. *An Nahar Daily* 23 Feb 2008

[131] British American Tobacco British American Tobacco’s views on the FCTC Protocol on Illicit Trade. November 2007. http://www.bat.co.uk/group/sites/uk__3mnfen.nsf/vwPagesWebLive/DO6ZYC4S/$FILE/medMD79RJU8.pdf?openelement (accessed 5 January 2008)

[132] Joossens L Raw M Turning of the tap: the real solution to cigarette smuggling. Int J Tuberc Lung Dis 2003;7:214–22 12661834

[133] Joossens L Chaloupka FJ Merriman D Issues in the smuggling of tobacco products. Jha P Chaloupka F, eds. Tobacco control in developing countries. Oxford: Oxford University Press, 2000:393–406

[134] Shepherd P Transnational corporations and the international cigarette industry. In *Profits, progress and poverty.** Case studies of international industries in Latin America*. Notre Dame, IN: University of Notre Dame Press, 1985

[135] Breton E Richard L Gagnon F Fighting a tobacco-tax rollback: a political analysis of the 1994 cigarette contraband crisis in Canada. J Public Health Policy 2006;27:77–99 16681189 10.1057/palgrave.jphp.3200060

